# Grape microbiome as a reliable and persistent signature of field origin and environmental conditions in Cannonau wine production

**DOI:** 10.1371/journal.pone.0184615

**Published:** 2017-09-11

**Authors:** Valerio Mezzasalma, Anna Sandionigi, Ilaria Bruni, Antonia Bruno, Gianni Lovicu, Maurizio Casiraghi, Massimo Labra

**Affiliations:** 1 Zooplantlab, Department of Biotechnology and Biosciences, University of Milano-Bicocca, Milan, Italy; 2 FEM2-Ambiente s.r.l., Milan, Italy; 3 Agricultural Research Agency of Sardinia (AGRIS), Sassari-Fertilia, Sassari, Italy; Universita degli Studi di Siena, ITALY

## Abstract

Grape berries harbor a wide range of microbes originating from the vineyard environment, many of which are recognized for their role in the must fermentation process shaping wine quality. To better clarify the contribution of the microbiome of grape fruits during wine fermentation, we used high-throughput sequencing to identify bacterial and fungi communities associated with berries and musts of Cannonau. This is the most important cultivar-wine of Sardinia (Italy) where most vineyards are cultivated without phytochemical treatments. Results suggested that microbiomes of berries collected at four different localities share a core composition characterized by Enterobacteriales, Pseudomonadales, Bacillales, and Rhodospirillales. However, any area seems to enrich berries microbiome with peculiar microbial traits. For example, berries belonging to the biodynamic vineyards of Mamoiada were rich in Bacillales typical of manure (i.e. *Lysinibacillus*, *Bacillus*, and *Sporosarcina*), whereas in the Santadi locality, berries showed soil bacteria such as Pasteurellales and Bacteroidales as well as Rhodospirillales and Lactobacillales which are commonly involved in wine fermentation. In the case of fungi, the most abundant taxa were Dothioraceae, Pleosporaceae, and Saccharomycodaceae, and although the proportion of these families varied among localities, they occurred ubiquitously in all vineyards. During vinification processes performed at the same wine cellar under controlled conditions and without using any yeast starter, more than 50% of bacteria groups of berries reached musts, and each locality had its own private bacteria signature, even if *Saccharomyces cerevisiae* represented the most abundant fungal species. This work suggests that natural berries microbiome could be influenced by pedoclimatic and anthropologic conditions (e.g., farming management), and the fruits’ microorganisms persist during the fermentation process. For these reasons, a reliable wine genotyping should include the entire holobiont (plant and all its symbionts), and bioprospecting activities on grape microbiota could lead to improved viticulture yields and wine quality.

## Introduction

To date, at least 5000–8000 grape cultivars showing particular traits (grape size, shape, color, and flavor) have been selected by viticulturists [1, 2]. Despite this huge diversity, variations in environmental conditions (i.e., soil composition, water management, and climate) and fermentation processes shape the contribution of these traits and modify the quality of the resulting wines. The identification of key environmental elements involved in the regional variation of grape and wine quality characteristics is a critical feature for improving wine production in terms of consumer preference and economic appreciation [3].

Several studies showed the effects of abiotic conditions on grapevine growth and fruit development, such as UV solar radiation [4], water availability [5], and nitrogen sources [6]. At the same time, biotic factors are also involved, since *Vitis vinifera* L. naturally hosts a reservoir of microorganisms [7–9] that interact with the plant and affect both the qualitative and quantitative scale of wine production.

The occurrence and effects of regional-specific microbiota in defining wine characteristics is a more controversial issue. Experimental analyses suggest that microbes colonizing berries could significantly affect grapevine and fruit health and development [10]. However, grapevine bacteria and yeasts also contribute to shaping phenotypic characteristics, such as flavour, colour, and sugar content [11], thus influencing the winemaking process as well [12, 13].

Recently, High-Throughput DNA Sequencing techniques (HTS) have being used to characterize bacterial communities of different grapevine plant portions, such as leaves and berries [14] and to assess the provenance in terms of plant portion and farming region of some microbial groups [15, 16]. Metagenomic analyses suggested that soil serves as a primary source of microorganisms with edaphic factors influencing the native grapevine microbiome, since the microbial communities of soils from the same viticultural region are quite heterogeneous [9]. Bokulich [15] showed that *Vitis* microbial biogeography is non-randomly associated with regional, varietal, and climatic factors across multiscale viticultural zones. Moreover, in 2016, the same research team [17] suggested a strong association involving grapevine microbiota, fermentation characteristics, and wine chemical composition.

Viticulturists are aware that the ground where plants grow imparts a peculiar metabolic trait on grapes and wine; this concept is usually referred to as *terroir*. In vineyards several variables could affect grape characteristics, such as soil composition and structure topography, climate conditions, and agricultural practices. Moreover, these conditions could also influence the plant microbiome. During vinification, other variables could act on the biotransformation of grape juice, such as the environmental conditions of the wine cellar [11, 17], as well as chemical and microbial processes involved in the fermentation processes. On the whole, wine characteristics (color, flavor, fragrance, sugar content) result from the complex interaction between abiotic and biotic elements occurring in two distinct environments: vineyards and wine cellars. To better clarify the diversity and dynamics of the microbiome belonging to these two environments and its role during wine production, we analyzed grapes and musts of cv. *Cannonau*, one of the most important black grape varieties cultivated in Sardinia (Italy).

Sardinia is the second largest Italian island located in the western Mediterranean to the south of Corsica between the Italian peninsula, Spain, and Tunisia. This island is ideally suited for viticulture [18], and it is characterized by a huge number of grapevine cultivars with different morphological and chemical characteristics [19]. Due to peculiar pedoclimatic conditions occurring in different parts the island, this model offers a great opportunity to study the relationships and changes occurring at both the environmental and grape microbiome.

In the present study, we investigated the bacterial and fungal microbiome of Cannonau berries cultivated from different localities and musts produced, under controlled conditions, at the same wine cellar. This presents the opportunity to track the microbial community from grapes to wine cellar. Specifically, the goals of our work were to: i) evaluate the microbial diversity at the vineyard level in response to different environmental conditions and farming management (e.g., biodynamic) of Sardinian localities ii) study the dynamics of microbial diversity from the vineyard to the wine cellar to estimate the impact of field bacteria on wine must.

## Materials and methods

### Samples collection and wine production

**Fig 1:**
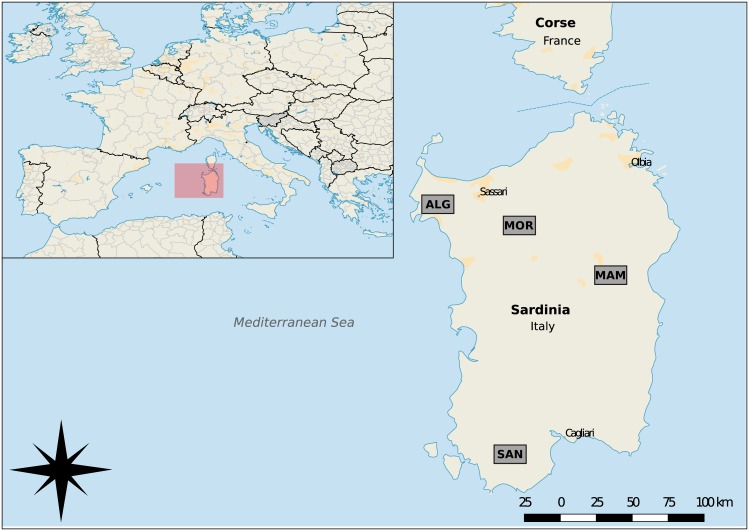
Map of Sardinia showing sampling vineyard localities. ALG (Alghero lat. 40.650 N, lon. 8.244 E), MOR (Mores lat. 40.517 N, lon. 8.806 E), SAN (Santadi lat. 39.090 N, lon. 8.793 E), MAM (Mamoiada lat. 40.222 N, lon. 9.309 E).

Given its pedoclimatic conditions and geographical isolation, Sardinian viticulture does not demand phytochemical treatments. For this reason, the island represents a suitable area to investigate the effect of natural field characteristics on the microbiome of grapes and therefore on the resulting must. To perform our experiments, we selected four Sardinian localities: Alghero (ALG), Mamoiada (MAM), Mores (MOR), and Santadi (SAN) (Fig 1).

Climatic and pedological conditions and the managed condition of vineyards for each locality are provided in Table 1.

**Table 1:** Vineyard characteristics: For each investigated locality, geographical, pedoclimatic, and farming systems information are provided.

Name	Geographical coordinates	Climatic condition	Pedological condition	Elevation (s.l.m.)	Orientation	Farming system
Alghero (ALG)	40.650°N, 8.244°E	Coastal Mediterranean plain	calcareous	70	70	conventional
Mamoiada (MAM)	40.222°N, 9.309°E	Mediterranean Mountain area	granites	760	760	biodynamic
Mores (MOR)	40.517°N, 8.806°E	Mediterranean interior plain	calcareous	280	280	conventional
Santadi (SAN)	39.090°N, 8.793°E	Mediterranean interior plain	schists-granites	230	230	conventional

During the harvest of 2015, mature grapes (20 degrees Brix) of the most diffused cultivar ‘*Cannonau*’ were collected as bunches bulks (100 berries) at each locality. One degree Brix is 1 gram of sucrose in 100 grams of solution and the scale is used as a proxy for grape maturation and fermentation progress. Sampling was performed in collaboration with specialized technicians of Agricultural Research Agency of Sardinia (AGRIS) at vineyards of the four localities. Although no specific authorization was required for sampling activities, vineyards owners gave permission to conduct the study on these sites. Field studies did not involve endangered or protected species. The collected Grape samples (G) were immediately frozen, shipped on ice, and stored at −80°C. These samples were used to characterize the berries microbiome (i.e. bacteria and yeast communities) of each locality.

To evaluate the effect of the environmental microbial community on must, we performed a wine-making process in controlled conditions starting from mature grapes collected from each locality. This was carried out at the experimental winery of AGRIS in a scale of 100 L per locality, without wine yeast starter and sulfur dioxide treatment. These conditions provide a natural fermentation process without any forced microbial selection [20]. This strategy is an emerging trend of winemaking to enhance natural interactions between microorganisms occurring during vinification [21]. To assess the characteristics and changes of must microbiome, we identified two phases based on the analysis of fermentation curves (which are usually conducted during winemaking process), namely initial must (iM) and end must (eM). The former has been collected about six hours after pressing, when the must shows the highest level of glucose (at least 20 degrees Brix) and the lowest level of ethanol (100 ml of iM for each locality). End must has been collected at 7 days after the winemaking process has started and when glucose level is lower than 2 degrees Brix and ethanol reach 12% v/v (100 ml of iM for each locality). In the case of iM samples, we were interested in evaluating the effect of the wine cellar on the original grape microbiome, whereas in the case of eM samples, we tested the dynamics of microbiome composition during the fermentation process. Each sample was stored at– 80°C.

### DNA extraction

Microbial biomass recovery from G samples was obtained starting from twenty berries randomly selected from each vineyard. These were thawed and placed in 500 mL sterile Erlenmeyer flasks. Berries were washed with 100 mL of isotonic solution (0.9% w/v NaCl) for 3 h with agitation at 150 rpm. The obtained cell suspensions were separated from the berries by centrifugation at 6,000 × g for 15 min. Pellets were stored at -20°C until DNA isolation.

In the case of must, microbial biomass was obtained from 10 mL of iM and eM samples. These were thawed and centrifuged at 6,000 × g for 15 min, washed three times in ice-cold isotonic solution. Pellets were stored at -20°C until DNA isolation. G, iM and eM samples were processed in duplicate.

Total genomic DNA were obtained from pellets using PowerSoil^™^ DNA Isolation Kit (MO BIO Laboratories, Carlsbad, CA, USA) following the manufacturer's instructions with modifications specific for wet soil samples.

Before libraries preparation, the obtained genomic DNA extracts were purified using Zymo Research DNA Clean and Concentrator-10 (Zymo Research, Irvine, CA, USA) to remove PCR inhibitors.

### Library preparation and sequencing

For each DNA sample, two independent DNA libraries, for bacteria and fungi, were prepared following Illumina guidelines (16S Metagenomic Sequencing Library Preparation, Part #15044223 Rev. B) with modifications. Bacterial V3 and V4 16S rRNA genes were amplified using primers S-D-Bact-0341-b-S-17 and S-D-Bact-0785-a-A-21 [22] with the addition of the Illumina overhang adapter sequences.

Fungal internal transcribed spacer (ITS) 1 loci were amplified with primers BITS and B58S3 [23], with the supplement of the Illumina overhang adapter sequences. Before amplification, DNA extracts were normalized by means of Quantitative real-time PCR (qPCR) Ct values with the same amplification primer pairs and the same protocols described by Bruno and colleagues [24, 25]. Finally, the obtained libraries were submitted to Polo d’Innovazione Genomica, Genetica e Biologia Società Consortile R.L. (POLO-GGB, Perugia, Italy) for Illumina paired-end library preparation, cluster generation, and 2 x 300-bp paired-end sequencing (MiSeq Reagent Kit v3) on an Illumina MiSeq instrument.

### Microbial composition and community structure analysis

Analysis of bacterial and fungal communities were performed using scripts of the QIIME pipeline [26]. Raw Illumina reads were paired and pre-processed using USEARCH merge pairs algorithm [27]. During the Quality filter step reads were filtered out if: 1) ambiguous bases were detected, 2) lengths were outside the bounds of 250 bp and/or 3) average quality scores over a sliding window of 40 bp dropped below 25.

Bacterial reads were then processed by VSEARCH 1.1.8 software version [28], which removed noise and chimeras prior to performing de novo clustering into OTUs at 97% sequence identity and discarding those OTUs represented by less than 75 sequences. The cluster centroid for each OTU was chosen as the OTU representative sequence. The taxonomic assignment of the representative sequences was carried out using the RDP Bayesian Classifier [29] against the SILVA SSU non-redundant database (version 119 release) adopting a consensus confidence threshold of 0.8. The RDP classifier was then used for the taxonomic assignment of OTUs.

Fungal reads were cleaned concerning the noise and the chimera using the same workflow adopted for bacteria reads. Before OTUs clustering, ITSx extractor [30] was used in order to filter non-fungi contaminant reads. De novo OTUs were calculated, as in the case of bacteria, using the VSEARCH cluster algorithm at 97% sequence identity with the cluster centroid for each OTU as the OTU representative sequence. The taxonomic assignment of the representative sequences was carried out using the RDP Bayesian Classifier against UNITE fungal database [31].

For both communities, a rarefaction table was calculated for each sample to determine the suitable sequencing depth that covers the extant microbial diversity.

The intra group diversity estimation (alpha diversity) was calculated using the number of observed OTUs and the Shannon index. Community analyses (beta-diversity) were performed with qualitative (Jaccard and unweighted UniFrac for fungi and bacteria respectively) and quantitative (Bray-Curtis and weighted UniFrac for fungi and bacteria respectively) distance metrics [32] using QIIME and *phyloseq* R package for statistical computing [33, 34]. Statistical significance among groups was determined by the ADONIS (permutation based ANOVA (PerMANOVA)) functions of the *vegan* R Package [35]. PerMANOVA Pairwise contrast was performed with R script [36].

The phylogenetic tree necessary to calculate UNIFRAC distances and based on the alignment of OTUs representative sequences was built using RAxML version 7.4.2 [37] with the GTRGAMMA model bootstrapping (1’000 replicates) best maximum likelihood tree inference. Multibar plots were generated with QIIME.

A Venn diagram was created with the online tool [38] by calculating the number of shared and unique OTUs in the different datasets.

## Results and discussion

### Sequence analysis

To characterize the microbial consortia associated with grapes and musts of Cannonau vineyards by HTS approach, a total of 1’600’000 and 5’000’000 quality-filtered sequences were obtained for the 16S rRNA and ITS1 marker, respectively. After the removal of low quality reads sequences failing the alignment or annotated as host or mitochondrial or chloroplast sequences, and singleton sequences, a total amount of 235’371 16S rRNA V3-V4 amplicon sequences belonging to the three fermentation steps (G, iM, eM) for 24 samples were considered for further bioinformatics analyses. These sequences had an average of 430 bp (ranging from 400 to 438 bp) with primer removal and clustered into 264 OTUs. Moreover, for the same set of samples and adopting the same procedure, a total of 216 ITS1 OTUs were supported by sequences with an average of 400 bp (ranging from 390 to 405 bp) (see for more details S1 Table).

### Bacteria and fungi OTU diversity

OTUs diversity of bacteria and fungi was analyzed separately and described in Table 2. Considering that the two replicates did not statistically differ for each sample (R2 P v>0), we decided to combine replicates to calculate alpha diversity. Concerning bacteria, the observed OTUs ranged from 50–113 for G samples, 46–103 for iM, and 67–70 for eM. Data suggests that all grape samples significantly differ from each other (see data in S1 Text) with the exception of ALG-MAM (p-value = 1). This was also confirmed by Shannon Indexes (Table 2) (see data in S1 Text). The diversity among localities decreases in must samples; in the case of iM samples, only those from SAN significantly differ from ALG and MAM, but they do not differ from those of MOR (p-value = 1). Significant differences were also detected between MOR iM and MAM iM (ANOVA F = -0.5552, p = 0.00006). Finally, the OTUs diversity among localities was reduced in the case of eM samples, and no significant differences were observed among musts belonging to all localities.

**Table 2:** Number of observed bacteria and fungi OTUs and related Shannon index for each sample typology at each sampling site.

Samples	Bacteria		Fungi	
	Observed OTU	Shannon Index	Observed OTU	Shannon Index
ALG G	55	2.175450	110	2.653449
ALG iM	59	2.229309	108	2.547885
ALG eM	70	2.217277	36	2.261021
MOR G	84	3.299958	117	2.269252
MOR iM	94	1.763944	69	1.290504
MOR eM	67	2.858682	26	2.464016
SAN G	113	2.366787	133	1.882989
SAN iM	103	2.101003	63	1.271993
SAN eM	69	2.764250	27	1.971678
MAM G	50	3.483344	94	1.892080
MAM iM	46	2.362264	52	2.254171
MAM eM	68	3.150848	21	2.391844

In the case of fungi, all the localities share the same trend: a high number of OTUs were detected in G samples and were lower in iM and eM samples. This could suggest that several fungal OTUs occurring at fruit levels do not reach the wine cellar or do not resist the wine fermentation process as previously shown by Gilbert and colleagues [39]. Moreover, fungi species occurring in wine cellars do not largely enrich the wine microbiome in terms of OTUs diversity [23].

**Fig 2:**
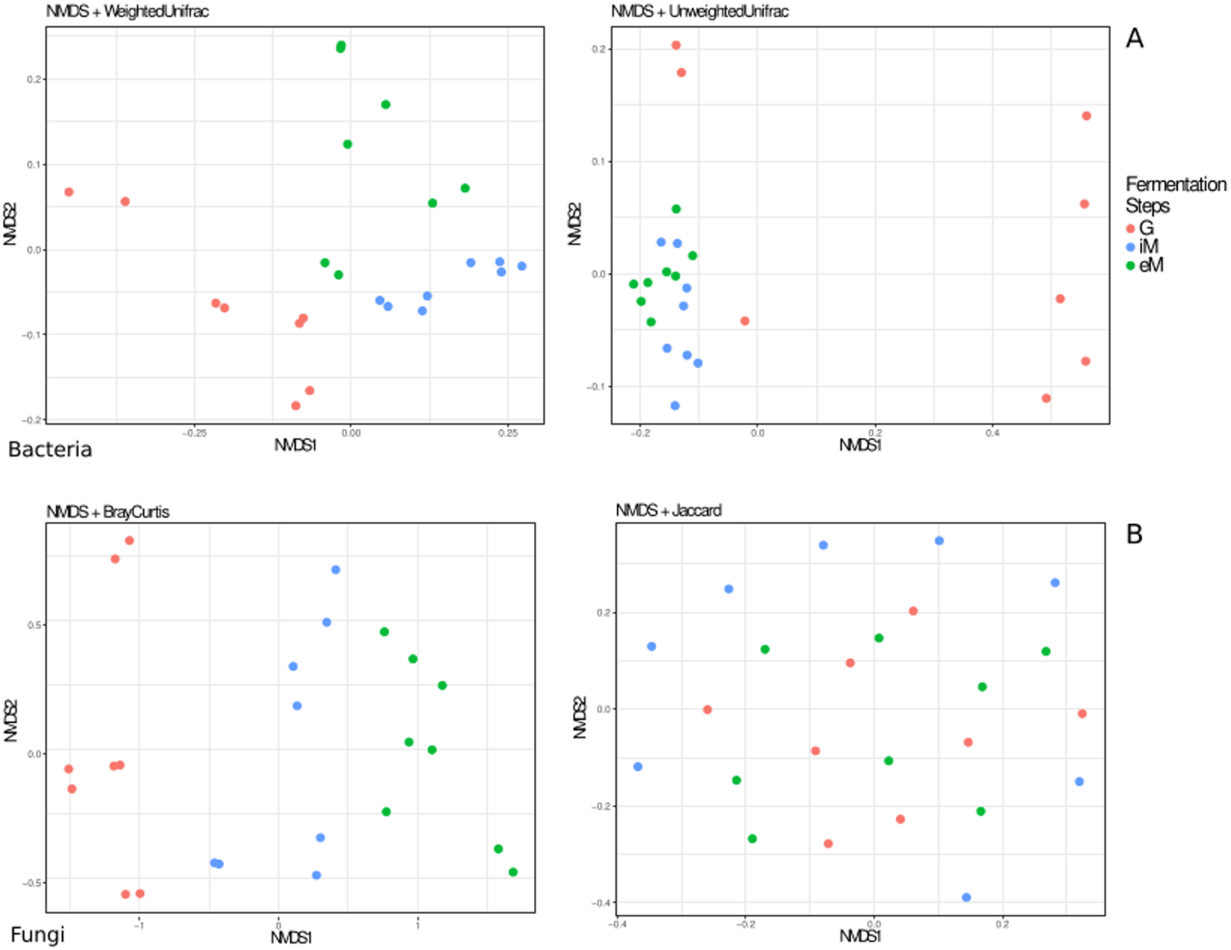
Microbial beta diversity at the four sampling localities. (A) Non-metric multidimensional scaling (NMDS) using unweighted and weighted UNIFRAC distance matrices as measures of beta diversity in bacterial communities. (B) NMDS using the Jaccard and Bray Curtis distance matrix coefficients to estimate the beta diversity of fungi communities. Red dots indicate grape samples (G), blue dots the initial must (iM) samples, and the green dots the end of must samples (eM).

To better estimate the microbial dynamics of grapes from fields to the wine cellar in the four study areas, we calculated the beta diversity. Fig 2A shows the Unweighted and Weighted UniFrac PCoA plot obtained by 16S rRNA data. As previously suggested, the two sample replicates showed good overlapping (PerMANOVA pairwise test results are shown in data in S2 Text). This confirms that the sampling strategy was adequate to depict the microbiome heterogeneity of the considered vineyard. Fig 2A showed clear differences between G and musts samples. Moreover, this analysis showed that the bacterial OTUs on fruit surfaces were very different among the four localities, and this information is well represented by the unweighted non-metric multidimensional scaling NMDS (ADONIS R^2^ = 0.34 p-value<0.001). The iM and eM samples clustered closer. These data support the hypothesis that most of the field bacteria taxa growing on fruit surfaces are not able to persist into the wine cellar environment. This condition could be related to the fact that most of these bacteria cannot resist the change from grape aerobic to must anaerobic conditions, as well as the antimicrobial effect of secondary metabolites and ethanol occurring in must.

However, during fermentation a different bacterial community arises in must probably originating from the wine cellar. These considerations agree with previous studies [40, 41] suggesting that different microorganisms diffused in the wine cellar play peculiar roles in the specific steps of the winemaking process [11] and that their mutual interaction could affect wine characteristics.

Fig 2B shows the beta diversity of fungi OTUs. Considering the relative abundance (Bray-Curtis analysis), a pattern similar to bacteria was observed, but in this case, differences between iM and eM are more consistent. This could further suggest that wine cellar environment influences the must microbiome during the fermentation process [42]. This hypothesis is particularly supported by looking at *S*. *cerevisiae*; although this yeast was not added as a commercial starter in the 4 analyzed musts, it also occurred in iM and eM samples. The wine cellar could represent a primary source of this yeast and probably the other non-*Saccharomyces* yeasts, however their occurrence and development in must is related to the complex relationships among microorganisms during different fermentation phases [11, 20, 43].

**Fig 3:**
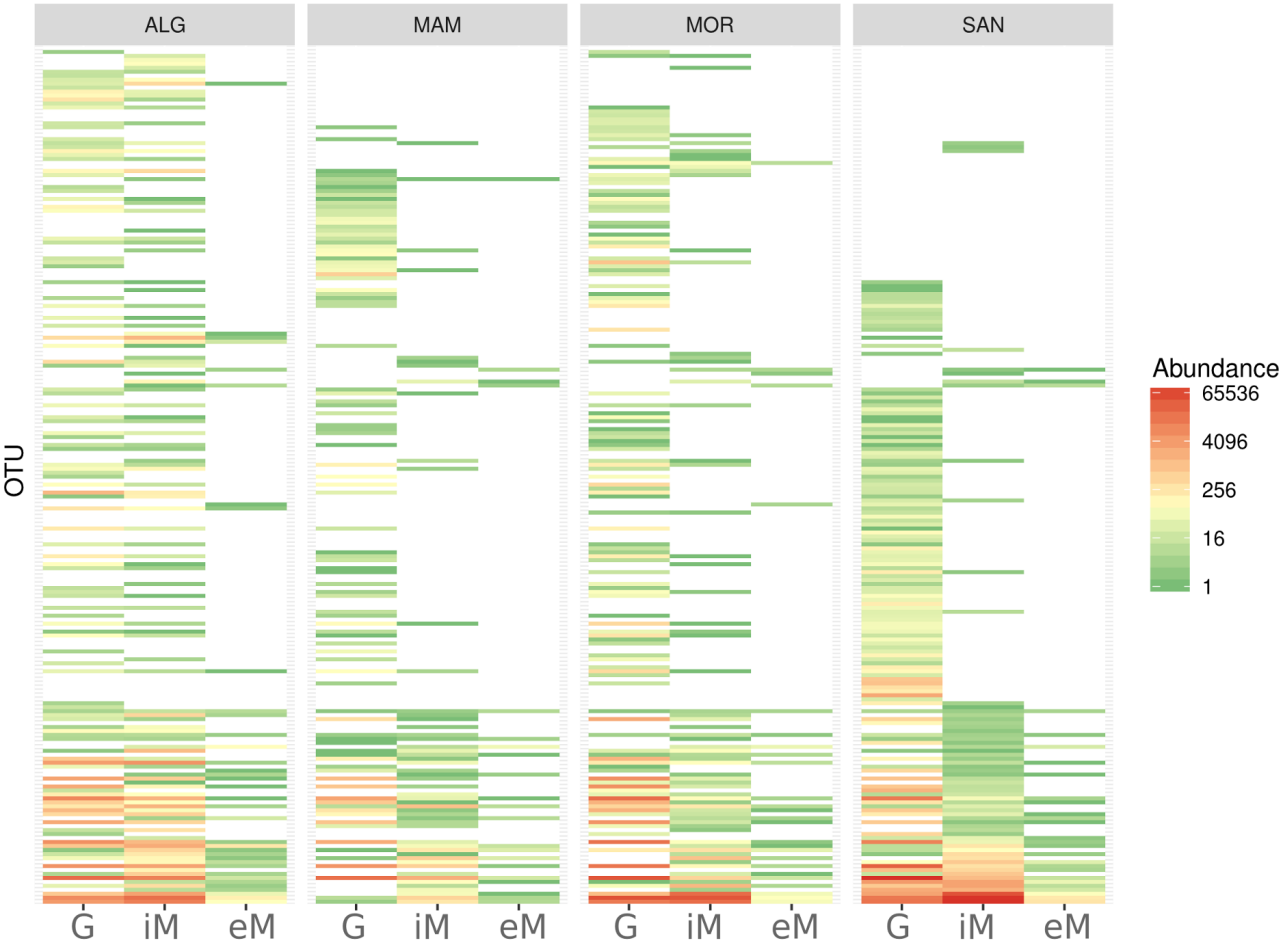
Heatmap of fungal OTUs. The figure shows the distribution of fungi OTUs for each sample typology (G, iM, and eM) at each sampling locality (ALG, MOR, SAN, and MAM).

To better illustrate the variation of fungi OTUs from field to wine cellar, we produced a heatmap analysis (Fig 3). This clearly showed a drop in the number of OTUs in the winery, from the initial phase of must (iM) and which became more appreciable in eM samples, as was also supported by the alpha diversity data for each locality (Table 2).

### Microbial taxonomy diversity

To understand the occurrence and the role of different microorganisms during the wine process and their origin, we analyzed the microbial taxonomy of the grape and must of *Cannonau* at different Sardinian localities.

The taxonomic assignment of sequences was performed using the RDP Classifier for both bacteria and fungi. PE sequences matching those in Silva and UNITE databases exceeding the 0.8 confidence threshold were classified and assigned to a taxonomic rank. Concerning bacteria, the 264 different OTUs were assigned to 13 predominant phyla, 44 orders, and 73 families. About of 80% of OTU were identified at the genus level. In the case of fungi, a total of 216 OTUs were detected and corresponded to 4 phyla, 35 orders, and 48 families.

**Fig 4:**
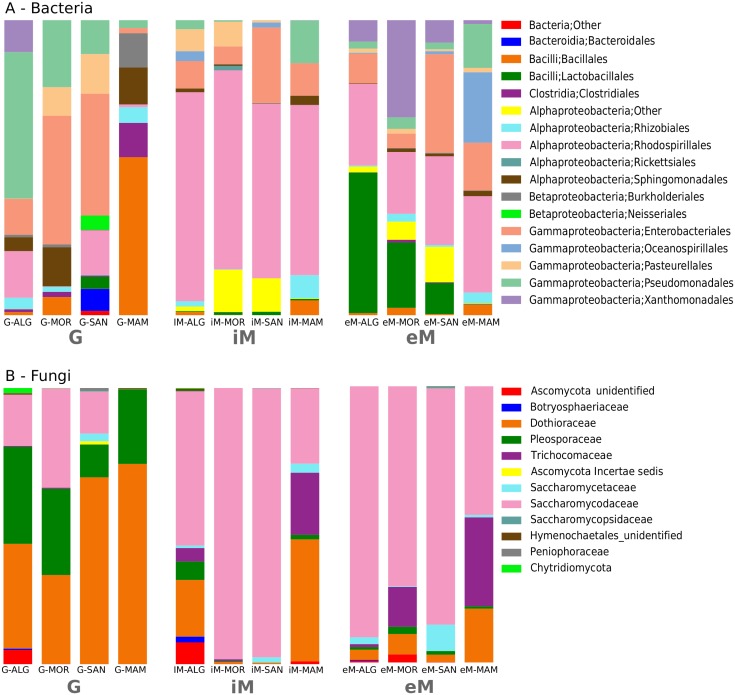
Distribution of bacterial and fungal assigned taxa. Relative abundance of bacterial orders (A) and fungi families (B) recovered in G, iM, and eM samples at each sampling locality. Where the assignment to the Order (bacteria) or Family (fungi) rank failed, the nearest taxonomic level with supported assignment has been reported.

Fig 4A describes the distribution of bacterial orders having a relative abundance > 0.01% determined by summing the counts derived from the two biological replicates for each sample distinguished in G, iM, and eM. Results confirm that G sample bacterial communities varied greatly across the four localities and were constituted predominantly by Enterobacteriales (19.5%), Pseudomonadales (17.5%), Bacillales (11.8%), and Rhodospirillales (8.8%). This finding agrees with data obtained by microbiome analysis performed on Grenache, one of the synonyms of Cannonau [44]. However, these four shared predominant bacteria orders should not be considered a private fingerprint of this genotype, because the same taxa were already detected on several other grapevine cultivars, such as Chardonnay, Cabernet, and Zinfandel [15]. These bacteria can be then considered a ‘common microbiome’ of vineyard soil, and they seem to not respond to pedological and environmental conditions [9, 16, 39]. The consistent presence of bacteria belonging to these orders on grape fruits could be explained by microbial migration through rain splash, winds, and insects as supported by Martins and co-workers [8], as well as by taking into account their adaptations to fruit characteristics [16]. Although the effect of these microorganisms on grape fruits and wine are unclear so far, we can conclude that a stable core microorganism of vineyards could be considered a basal biotic element able to influence different grape organs and any plant growth stages [45]. A second biotic element consists of the ‘peculiar microbial’ community that is characteristic of each vineyard and is influenced by environmental conditions and anthropogenic factors. As shown in Fig 4A, all 4 analyzed vineyards have different microbiomes at the berries level. However, samples of SAN and MAM localities showed the most peculiar microbial diversity in comparison to other localities. In SAN G samples, a consistent occurrence of Rhodospirillales (14.5%), Pasteurellales (13.1%), Bacteroidales (7.6%) was detected. Members of these taxa have been previously described in the vineyard soil microbiome. Specifically, Zarraonaindia [9] detected them in five vineyards in Long Island (NY, US) characterized by granitic soil. The acidic conditions of the soil in SAN, due to the occurrence of schists-granites soil (Table 1), could support the development of similar traits to the microbiome of Long Island. Therefore, ‘peculiar’ berries microbiome could be directly influenced by soil characteristics, such as pH and soil nutritional resources [46].

Another characteristic trait of the SAN G microbiome is the modest presence of Acetobacteraceae (Rhodospirillales 14.5%) and Lactobacillaceae (1.8%), which are typically involved in the glucose fermentation of must. The occurrence of these bacteria could suggest an over-ripening of grape fruits in this vineyard, which it is generally accompanied by fruit softening and partial degradation of exocarp resulting in leakage of the grape’s sugary juice. However, all sampling was performed at 20 degrees Brix, thus excluding over-ripening. Another possibility could be related to the damage of fruits. SAN samples were characterized by a relevant percentage (i.e. > 30%) of berries damaged by insects, probably belonging to Hymenoptera (AGRIS Personal communication). This event causes the fruit juice, rich in sugars content, to come out and promotes bacterial growth. In this case, external biologic factors might have influenced the berries microbiome with possible consequences on the final wine, because these microorganisms are able to actively and prematurely begin the fermentation process.

Concerning MAM samples, the collected fruits were dominated by Bacillales (32%) including members of *Lysinibacillus*, *Bacillus* (Bacillaceae), and *Sporosarcina* (Planococcaceae). These bacteria were found in the manure of cows, pigs, and poultry [47, 48, 49], and their occurrence in MAM could be explained by considering the biodynamic farming system applied in this vineyard. Specifically, in MAM a cow horn filled with manure was buried for maturation and subsequently was activated with water during the spring (dynamization), and the resulting product was sprayed into the field. This strategy is declared to improve soil quality, as well as enhancing plant growth and resistance to pathogens. Recent works suggested that the effect of biodynamic management could also be explained by the modification of the plant microbiome [7, 50, 51], because bacteria can act as biological disease suppression agents and could also stimulate plant growth and have an effect on plant health.

The biodynamic practice could also explain the conspicuous presence of Clostridia bacteria (4.5%) with members of Peptostreptococcaceae and Clostridiaceae. Both of these bacteria have also been detected in manure [52] and could support the beneficial effect on plant growth as well.

We conclude that in MAM the characteristic traits of the fruits microbiome are largely influenced by agricultural management and that bacteria originally related to animal manure is also able to grow on grape fruits. However on MAM G samples, we also observed members of Burkholderiales (i.e. *Massilia* sp.) and Rhizobiales (*Rhizobium* sp.) which are typically diffused in soil rich in organic matter fractions. Also, these bacteria are important players for viticulture soils, since they are able to promote plant growth [53]. On the whole, we can hypothesize that the berries microbiome characteristics reliably reflects the soil fertility of the vineyard. This is also evident in the plant of MAM vineyards, which were highly vigorous in vegetative organs, such as leaves and branches.

Concerning ALG and MOR vineyards, G samples shared the same microbial orders with a large abundance of Pseudomonadales and Enterobacteriales. This condition probably resulted from similarities in pedoclimatic characteristics: e.g. the same localities showed calcareous soil. However, some differences between these localities were detected: in MOR G samples, members of Pasteurellales were detected, whereas in ALG G samples, Xanthomonadales occurred.

Concerning the yeast diversity of G samples, the most abundant taxa were Dothioraceae (*Aureobasidium*, 49.86%), Pleosporaceae (*Alternaria*, 18.43%; *Pleospora*, 6.63%), and Saccharomycodaceae (*Hanseniaspora*, 17.63%). Although the proportion of these families changed among localities, their presence was ubiquitous in all vineyards. Berries of MAM were characterized by the absence of Saccharomycodaceae, whereas the group Saccharomycetaceae was detected in SAN, but not in the others localities.

The taxonomic diversity of musts was more moderate than that of G samples with a consistent relative abundance of Rhodospirillales (from 67.2% in ALG to 50.3%% in MAM), represented by the genera *Gluconobacter* and *Gluconacetobacter*, involved in the initial steps of the fermentation process. Members of Pseudomonadales, Bacillales, and Enterobacillales occurring in G samples, dropped in musts, because they did not have the ability to grow during the wine fermentation processes [10]. Our analysis suggests moderate differences of the microbiome among samples coming from the four localities. We underline that all the collected berries were processed at the same experimental wine cellar; therefore, all musts could be influenced by the same wine cellar’s microbiome. Moreover, the winemaking process was performed under the same chemical-physical parameters. For these reasons, the only appreciable differences lay in the persistence of microorganisms deriving from the vineyard, like the Rhizobiales and Pseudomonadales (i.e., *Acinetobacter*) in MAM samples.

In eM samples, the microbiome was more variable among the samples rather than in iM samples. We detected *Gluconobacter* and *Gluconacetobacter* (Rhodospirillales) involved in must fermentation as well as *Lactobacillus* (Bacillales) involved in malolactic fermentation. In general, malolactic fermentation is most often performed shortly after the end of the alcoholic fermentation, and for this reason, it is active during eM and not in iM. In the MAM eM samples, we did not detect Lactobacillales, but there were some species described in the must, such as bacteria belonging to *Carnomonas* (Oceanospirillales) [54].

In the case of fungi, the most abundant species in must was represented by *S*. *cerevisiae*. Although this organism did not occur on the grape surface, starting from the first fermentation steps, it became dominant in must due to its higher fermentative ability, growth rate, and tolerance to ethanol [55]. The primary source of this yeast is crush equipment and barrel room surfaces [23], and this explains its presence in our must samples treated without any use of commercial starter. Starting from the first phases of fermentation, *S*. *cerevisiae* supplants the various non-*Saccharomyces* yeasts and modifies the must’s characteristics with consequences on the whole microbiome [23]. To better assess the peculiar yeasts occurring in the iM and eM samples of the four localities, the bar chart of Fig 4B was computed without *S*. *cerevisiae* OTUs. Data suggest that the second abundant yeast family is represented by Saccharomycodaceae with *Hanseniaspora uvarum* and other fermentative yeasts, such as members of Trichocomaceae (i.e. *Aspergillus* spp. in MOR and MAM) and Saccharomycetaceae (i.e. *Candida* spp. in SAN). Although these yeasts were less represented than *S*. *cerevisiae*, they are important microbial actors involved in wine fermentation, and they are able to modify wine aroma and other organoleptic characteristics through the production of a greater range of sensory-active compounds. For example, *Hanseniaspora uvarum* products 2-phenylethyl acetate, which contributes to the rose, honey, fruity and flower aromas of wines [56 57, 58]. The origin of these yeasts is partially unclear. Some of these are detected on grapes [10], while others could be resident microorganisms of wine cellar [42]. Considering that in our study all the wine juices were fermented in the same wine cellar, we could hypothesize that differences in yeasts occurring in musts could derive from the field. Although these yeasts were not detected on the berries surfaces (e.g., *Hanseniaspora uvarum*, *Aspergillus* spp. and *Candida*), we cannot exclude their presence as spores not detectable by our NGS sequencing analysis, but they may become appreciable in must where they are able to germinate and proliferate. Nowadays, it is critical not only to define which microbes contribute to create a high-quality wine, but also how their metabolisms can influence wine organoleptic characteristics. Integrated databases, based on HTS and biochemical data, will permit in the very next future to analyze in depth the effects of a certain microbiome on metabolome. For example the WineSeq^®^ platform (Biome Makers, Inc.) [59] revealed that the occurrence in G samples of some yeasts could have important effects on wine quality and human health. This is the case of the detected *Hanseniaspora* sp. yeast is related to a potential sensory profile enhancement on wine flavour.

### From field to wine cellar

Many studies suggest that the grapevine’s microbiome influences the plant’s physiology, and it can then also determine some aspects of the secondary metabolites’ profile shown by fruits [11, 17]. A challenging issue to address is the impact of the berries’ indigenous microbiota on wine fermentation and the consequent effect on the sensory complexity of wines [20]. Yeasts play important roles during the alcoholic fermentation step and also have a significant impact on wine quality. However, in our experimental conditions, bacteria represented the most relevant elements of differentiation among localities (108 private OTUs out of 176 detected on G samples); therefore, we studied the persistence of the characteristic field microbiome of different localities in must samples. We underline that although bacteria are not the main driving force shaping wine characteristics and quality, they do have a significant effect on the final product. For example, lactic acid bacteria are known to convert L-malic acid to lactic acid through MLF and to impart flavor complexity, while acetic acid bacteria produce acetic acid, which is a key factor in wine spoilage. Similarly, we can expect that the bacteria occurring on Cannonau berries at different localities may play a key role in must fermentation and wine quality. However, only the investigation of bacteria communities’ dynamics during Cannonau fermentation could assess the influence of each microbial group on wine characteristics.

**Fig 5:**
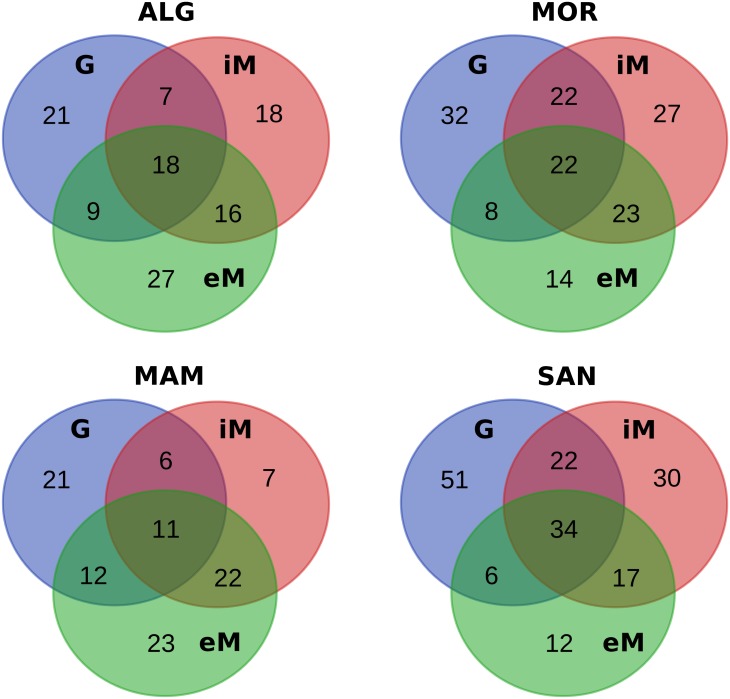
Venn diagram of shared OTUs. The figure shows the number of shared OTUs among sample typologies belonging to the four sampling localities.

**Fig 6:**
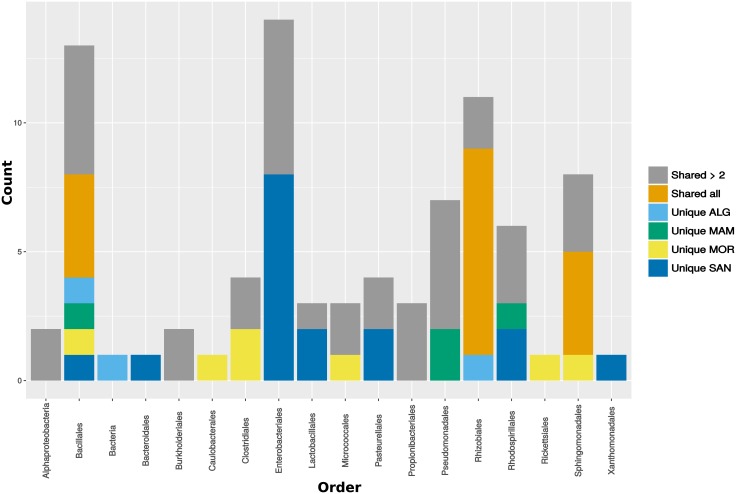
Barplot showing the distribution of unique bacterial OTUs (*y* axis) in G samples. On the *x* axis, bacterial orders are reported. Gray bars indicate the number of OTUs belonging to a specific Order shared by more than two localities. Orange bars indicate how many OTUs are shared among all four sampling localities. The other colors indicate the number of unique OTUs belonging to different orders.

A Venn graph (Fig 5) shows that more than 50% of G OTUs reach the wine cellar in all localities. As suggested by Bokulich [17], vineyard-specific microbial signatures diminished during fermentation (Figs 4A, 4B and 5) as the growth of fermentative organisms reshaped the community structure, richness, and diversity of the wines. However, our data showed that more than 50% of shared OTUs between G and iM persisted in the eM phase. These bacteria could modify wine traits [11, 17, 60] not only at the field level, but also by active metabolism in must [11, 46, 53]. Fig 6 shows that those OTUs shared by field and wine cellar environments (Venn center diagram) correspond to several bacterial orders and most of these are shared by two localities at least (Fig 6). In any case, each locality has its own private OTUs. Among the investigated localities, SAN showed the highest number of unique microbial traits (see S2 Table) and representatives of Enterobacteriales, Pasteurellales, Rhodospirillales, and Lactobacillales reach the wine cellar. As previously discussed, some of these bacteria are most active during fermentation, such as *Gluconobacter* (Rhodospirillales) and *Lactobacillus* (Lactobacillales). A few OTUs were detected as unique fingerprints of ALG and MAM, but some of these were very active in must fermentation, such as the *Acetobacter* (Rhodospirillales) detected in MAM. Finally, a microbial fingerprint of MOR grape and must was characterized by several orders including the member of Caulobacteriales and Clostridiales with genera involved in plant growth stimulation [61]. Their role in wine fermentation is still unclear, but we cannot exclude that these bacteria could be able to modify some wine’s metabolites. In conclusion, we can affirm that microbiomes found at the four investigated Sardinian localities can have an impact on fruit and, must. In addition, metabolism of different microorganisms could positively enhance some wine flavor traits, but also emphasize negative organoleptic characteristics.

## Conclusions

In this study, we demonstrated a distinct microbial composition of *Cannonau* fruits from different Sardinian localities with consequent effects also on the musts’ microbiome. To date, the role of grapevine microbes in the field has been largely ignored, with the only exception of microbial pathogens, mainly because the available technologies did not exist, and this prevented examining the community structure of the multitudes of bacterial and fungal species associated with each plant at any real depth or breadth [39]. Thanks to the HTS approach, we can now evaluate the microbial community of the grape and wine also in response to different environmental conditions and farming practices [46]. Probably, in the very next future, this technology will be also used to deeply investigate viruses and phytoplasma that largely influence vineyard sector [62].

Emerging work clearly show that pedoclimatic conditions could affect wine characteristics not only due to the abiotic characteristics (i.e. soil, sun exposition), but also at the grape microbiome that is able to influence plant growth and development [10]. Bokulich [15] demonstrated the existence of regional microbiome fingerprints in California vineyards; Portillo [44] showed that several environmental variables, such as vineyard altitude and the geographical orientation of the plant could also affect the grape microbiome. Our study confirms that pedoclimatic characteristics could modify the fruit microbiome and underlines that agricultural practices, such as biodynamics, as well as the occurrence of opportunistic insects, such as hymenopterans, can have a consistent effect on the bacterial communities of berries and corresponding must. These results suggest that the role of the field environmental microbiome is not limited to promoting grape fruit maturation and enhancing the occurrence of some secondary metabolites strictly related to wine color and flavor, but it is also an important source of microorganisms that are able to influence wine fermentation and metabolic composition.

Characteristics of the cultivars’ genotype play an important role in viticulture, so starting from the 1990’s, DNA fingerprinting approaches were used to identify synonymous cultivars and to unmask incorrect attributions. Our team [63], demonstrated a complete genetic identity between Cannonau and Spanish Grenache by using SSR markers. However, the microbiomes of these cultivars are very different [44], and appreciable differences were also observed among Grenache localities. This finding suggests that the value of the cultivar genotype is somewhat relative. A reliable genotyping should include the entire holobiont (i.e. the plant and all its symbionts [64]) of a specific Cannonau or Garnacha plant. The study of the grapevine microbiome does not represent a simple element of the product’s traceability and identity. We should consider that bioprospecting activities on grape microbiota could led to the discovery of several species with positive enological properties, as recently documented by the WineSeq^®^ project [59]. Occurrence and abundance of these species could be easily monitored by using conventional cultivation strategies and target PCR approaches (Real-Time and Digital PCR) to be used for improving wine quality, to enhance immune capability, and reduce the use of agrochemicals. Nowadays, only an exhaustive knowledge about the vineyard, the winery and their inhabitants could permit real advancements in management activities aimed towards a better sustainable system without any loss in terms of yields and product quality.

## Supporting information

S1 Text ANOVA result on alpha diversity values for 16S and ITS1. The ANOVA and Pairwise Post-hoc test results performed for each locality considering the three fermentation steps.
(DOCX)

S2 Text ADONIS test results. Results of ADONIS test performed to explore beta diversity patterns.
(DOCX)

S1 Table Run performance detail Table. (DOCX)

S2 Table Table of unique OTUs distribution. Dataframe of unique OTUs distribution for localities based on Venn diagrams analysis.
(XLSX)
