# Supplementary material for: Grape microbiome as a reliable and persistent signature of field origin and environmental conditions in Cannonau wine production
Source: PLoS One. 2017 Sep 11;12(9):e0184615. doi: 10.1371/journal.pone.0184615 (PMC5593190; doi:10.1371/journal.pone.0184615)

**Significant alpha diversity interactions considering Observed OTUs**

diff         lwr         upr      p adj

**Among G different Localities**

**16S**

G:Mores-G:Alghero    2.750000e+01   0.1976667  54.802333 0.0477842
G:Santadi-G:Alghero   5.400000e+01  26.6976667  81.302333 0.0001744

G:Mores-G:Mamoiada  2.850000e+01   1.1976667  55.802333 0.0379752

G:Santadi-G:Mores   2.650000e+01  -0.8023333  53.802333 0.0600727

G:Santadi-G:Mamoiada  5.500000e+01  27.6976667  82.302333 0.0001453

All G sample are significantly different from each other, the only exception is ALG and MAM (p-value = 1).

**its1**

G:Mores-G:Mamoiada 22.0 1.659009 42.340991 0.0299666

G:Santadi-G:Mamoiada 33.5 13.159009 53.840991 0.0010065

**Among iM different Localities**

**16S**

iM:Santadi-iM:Alghero 4.450000e+01 17.1976667  71.802333 0.0011070
iM:Mores-iM:Mamoiada 3.650000e+01 9.1976667  63.802333 0.0061444

iM:Santadi-iM:Mamoiada 5.550000e+01 28.1976667  82.802333 0.0001327

**its1**

iM:Mamoiada-iM:Alghero -52.0 -72.340991 -31.659009 0.0000123

iM:Mores-iM:Alghero -39.0 -59.340991 -18.659009 0.0002371

iM:Santadi-iM:Alghero -42.0 -62.340991 -21.659009 0.0001134

**Among eM different Localities**

There are no differences among eM that come from different cultivation sites for both markers.

**Considering same locality among G-iM-eM**:

**##16S**

**ALG**

There are no significant differences

**MOR**

There are no significant differences

**SAN**

eM:Santadi-G:Santadi      -4.050000e+01 -67.8023333 -13.197667 0.0025636

iM:Santadi-eM:Santadi    3.450000e+01   7.1976667  61.802333 0.0096204
**MAM**

There are no significant differences

**##its1**

**ALG**
eM:Alghero-G:Alghero -66.0 -86.340991 -45.659009 0.0000009

iM:Alghero-G:Alghero -1.5 -21.840991 18.840991 1.0000000

iM:Alghero-eM:Alghero 64.5 44.159009 84.840991 0.0000012

**MOR**

eM:Mores-G:Mores -82.0 -102.340991 -61.659009 0.0000001

iM:Mores-G:Mores -48.5 -68.840991 -28.159009 0.0000258

iM:Mores-eM:Mores 33.5 13.159009 53.840991 0.0010065

**SAN**

eM:Santadi-G:Santadi -93.0 -113.340991 -72.659009 0.0000000

iM:Santadi-G:Santadi -63.0 -83.340991 -42.659009 0.0000015

iM:Santadi-eM:Santadi 30.0 9.659009 50.340991 0.0026950

**MAM**

eM:Mamoiada-G:Mamoiada -64.0 -84.340991 -43.659009 0.0000013

iM:Mamoiada-G:Mamoiada -39.5 -59.840991 -19.159009 0.0002092

iM:Mamoiada-eM:Mamoiada 24.5 4.159009 44.840991 0.0138796

**Significant alpha diversity interactions considering Shannon Index**

diff         lwr         upr      p adj

**Among G different Localities**

**##16S**

G:Mamoiada-G:Alghero 1.163710802  0.90875985  1.418661755 0.0000000

G:Mores-G:Alghero  1.110571214  0.85562026  1.365522167 0.0000000

G:Santadi-G:Mamoiada -0.925280294 -1.18023125 -0.670329341 0.0000003

G:Santadi-G:Mores  -0.872140706 -1.12709166 -0.617189753 0.0000005

All G sample are significantly different from each other, the only exception is MOR=MAM and SAN=ALG (p-value = 1)

**##its1**

G:Mamoiada-G:Alghero -0.757906867 -0.94694729 -0.568866441 0.0000001

G:Mores-G:Alghero -0.395509932 -0.58455036 -0.206469506 0.0000993

G:Santadi-G:Alghero -0.762850078 -0.95189050 -0.573809652 0.0000001

G:Mores-G:Mamoiada 0.362396935 0.17335651 0.551437361 0.0002374

G:Santadi-G:Mores -0.367340146 -0.55638057 -0.178299720 0.0002078

**Among iM different Localities**

**##16S**

iM:Mores-iM:Alghero      -0.437990490 -0.69294144 -0.183039537 0.0006802

iM:Mores-iM:Mamoiada     -0.555282653 -0.81023361 -0.300331700 0.0000659

iM:Santadi-iM:Mores       0.342933945  0.08798299  0.597884898 0.0058456

Mores is the locality that maintain an isolation level.

**##its1**

iM:Mamoiada-iM:Alghero -0.304900154 -0.49394058 -0.115859728 0.0012181

iM:Mores-iM:Alghero -1.262674217 -1.45171464 -1.073633791 0.0000000

iM:Santadi-iM:Alghero -1.277719280 -1.46675971 -1.088678854 0.0000000

iM:Mores-iM:Mamoiada -0.957774063 -1.14681449 -0.768733637 0.0000000

iM:Santadi-iM:Mamoiada -0.972819126 -1.16185955 -0.783778699 0.0000000

**Among eM different Localities**

**##16S**

eM:Mamoiada-eM:Alghero  0.834182690  0.57923174  1.089133643 0.0000008

eM:Mores-eM:Alghero     0.581140835  0.32618988  0.836091788 0.0000412

**##its1**
eM:Santadi-eM:Alghero -0.279319142 -0.46835957 -0.090278716 0.0026526
eM:Santadi-eM:Mamoiada -0.355149535 -0.54418996 -0.166109109 0.0002892

eM:Santadi-eM:Mores -0.420928151 -0.60996858 -0.231887724 0.0000525

**Considering same locality among G-iM-eM:**

**##16S**

**ALG**

There are no significant differences

**MOR**

eM:Mores-G:Mores -0.466189949 -0.72114090 -0.211238996 0.0003759

iM:Mores-G:Mores -1.490951796 -1.74590275 -1.236000843 0.0000000

iM:Mores-eM:Mores -1.024761847 -1.27971280 -0.769810894 0.0000001

**SAN**

eM:Santadi-G:Santadi 0.355425427 0.10047447 0.610376380 0.0043508

iM:Santadi-G:Santadi -0.275877146 -0.53082810 -0.020926193 0.0298689

iM:Santadi-eM:Santadi -0.631302573 -0.88625353 -0.376351620 0.0000173

**MAM**

iM:Mamoiada-G:Mamoiada -0.988808731 -1.24375968 -0.733857778 0.0000001

iM:Mamoiada-eM:Mamoiada -0.722521049 -0.97747200 -0.467570096 0.0000041

**##its1**

**ALG**

eM:Alghero-G:Alghero -0.421837257 -0.61087768 -0.232796831 0.0000514

iM:Alghero-eM:Alghero 0.324306862 0.13526644 0.513347289 0.0006891

**MOR**

iM:Mores-G:Mores -0.964694680 -1.15373511 -0.775654254 0.0000000

iM:Mores-eM:Mores -1.079976364 -1.26901679 -0.890935937 0.0000000

**SAN**

iM:Santadi-G:Santadi -0.612399597 -0.80144002 -0.423359170 0.0000009

iM:Santadi-eM:Santadi -0.674093276 -0.86313370 -0.485052849 0.0000003

**MAM**

eM:Mamoiada-G:Mamoiada 0.411900003 0.22285958 0.600940429 0.0000657

iM:Mamoiada-G:Mamoiada 0.355476318 0.16643589 0.544516744 0.0002867


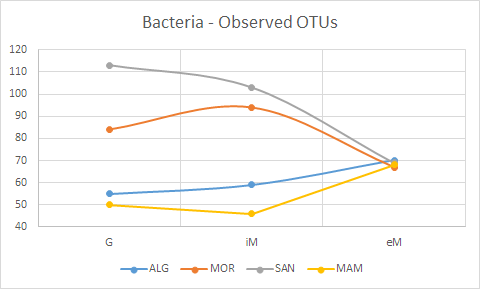

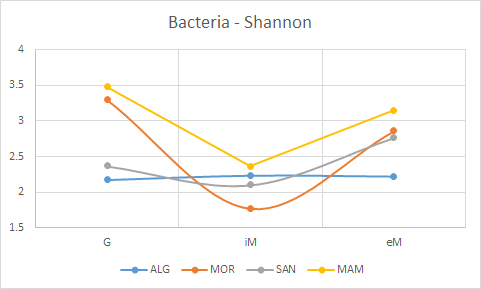


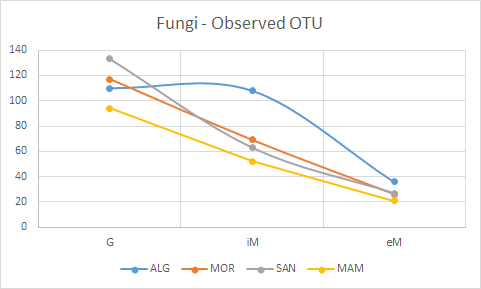

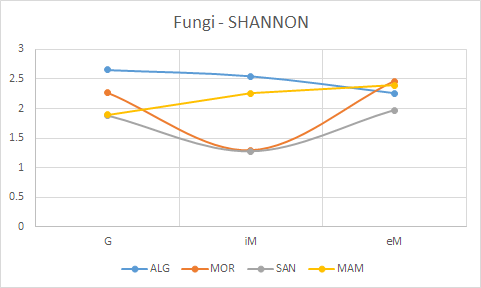

Supplement: S1 Text — The ANOVA and Pairwise Post-hoc test results performed for each locality considering the three fermentation steps. (DOCX) [file pone.0184615.s001.docx]
