# Supplementary material for: Grape microbiome as a reliable and persistent signature of field origin and environmental conditions in Cannonau wine production
Source: PLoS One. 2017 Sep 11;12(9):e0184615. doi: 10.1371/journal.pone.0184615 (PMC5593190; doi:10.1371/journal.pone.0184615)
Supplement: S2 Text — Results of ADONIS test performed to explore beta diversity patterns. (DOCX) [file pone.0184615.s002.docx]

Type= fermentation steps

Cultivation_Site= ALG, MOR, SAN, MAM

**16S**

##unweighted unifrac distance matrix

Call:

adonis(formula = unweight.dist ~ Type * Cultivation_Site, data = df.wine)

Permutation: free

Number of permutations: 999

Terms added sequentially (first to last)

Df SumsOfSqs MeanSqs F.Model R2 Pr(>F)

Type 2 1.1219 0.56096 11.7381 0.34629 0.001 ***

Cultivation_Site 3 0.6617 0.22058 4.6156 0.20425 0.001 ***

Type:Cultivation_Site 6 0.8827 0.14711 3.0783 0.27245 0.001 ***

Residuals 12 0.5735 0.04779 0.17701

Total 23 3.2398 1.00000

---

Signif. codes: 0 ‘***’ 0.001 ‘**’ 0.01 ‘*’ 0.05 ‘.’ 0.1 ‘ ’ 1

##weighted unifrac distance matrix

Call:

adonis(formula = wine.dist ~ Type * Cultivation_Site, data = df.wine)

Permutation: free

Number of permutations: 999

Terms added sequentially (first to last)

Df SumsOfSqs MeanSqs F.Model R2 Pr(>F)

Type 2 0.95041 0.47520 115.181 0.48852 0.001 ***

Cultivation_Site 3 0.42870 0.14290 34.637 0.22036 0.001 ***

Type:Cultivation_Site 6 0.51686 0.08614 20.880 0.26567 0.001 ***

Residuals 12 0.04951 0.00413 0.02545

Total 23 1.94548 1.00000

---

Signif. codes: 0 ‘***’ 0.001 ‘**’ 0.01 ‘*’ 0.05 ‘.’ 0.1 ‘ ’ 1

**ITS1**

**##**jaccard distance matrix

Call:

adonis(formula = jaccard.dist ~ Type * Cultivation_Site, data = df.wine)

Permutation: free

Number of permutations: 999

Terms added sequentially (first to last)

Df SumsOfSqs MeanSqs F.Model R2 Pr(>F)

Type 2 -1.08959 -0.54479 -2.48194 -0.82683 0.999

Cultivation_Site 3 -0.20219 -0.06740 -0.30705 -0.15343 0.954

Type:Cultivation_Site 6 -0.02447 -0.00408 -0.01858 -0.01857 0.987

Residuals 12 2.63404 0.21950 1.99883

Total 23 1.31779 1.00000

>

##Bray-Curtis distance matrix

Call:

adonis(formula = bray.dist ~ Type * Cultivation_Site, data = df.wine)

Permutation: free

Number of permutations: 999

Terms added sequentially (first to last)

Df SumsOfSqs MeanSqs F.Model R2 Pr(>F)

Type 2 2.3259 1.16293 112.118 0.47443 0.001 ***

Cultivation_Site 3 1.5923 0.53077 51.171 0.32480 0.001 ***

Type:Cultivation_Site 6 0.8598 0.14331 13.816 0.17539 0.001 ***

Residuals 12 0.1245 0.01037 0.02539

Total 23 4.9025 1.00000

---

Signif. codes: 0 ‘***’ 0.001 ‘**’ 0.01 ‘*’ 0.05 ‘.’ 0.1 ‘ ’ 1
