# Supplementary material for: Grape microbiome as a reliable and persistent signature of field origin and environmental conditions in Cannonau wine production
Source: PLoS One. 2017 Sep 11;12(9):e0184615. doi: 10.1371/journal.pone.0184615 (PMC5593190; doi:10.1371/journal.pone.0184615)
Supplement: S1 Table — (DOCX) [file pone.0184615.s003.docx]

Detailed yields of the two run (16S and ITS1): reads, OTUs obtained

| **16S** | quality-filtered sequences +singleton | no Eucariota, chloroplast, mitocondria and Unclassified < 0.1% | no Eucariota, chloroplast, mitocondria and Unclassified < 0.1% + Only Villasor wine making |
| --- | --- | --- | --- |
| Num samples | 32 | 32 | 24 |
| OTU | 702 | 264 | 264 |
| total count | 1583562 | 304829 | 235371 |
| Min reads x sample | 4816 | 176 | 180 |
| Max reads x sample | 117534 | 79666 | 79666 |

| **ITS1** | quality-filtered sequences +singleton | No Saccharomices cerevisiae | No Saccharomices cerevisiae + Only Villasor wine making |
| --- | --- | --- | --- |
| Num samples | 32 | 32 | 24 |
| OTU | 222 | 216 | 216 |
| total count | 5162064 | 1303420 | 1041606 |
| Min reads x sample | 14638 | 104 | 104 |
| Max reads x sample | 328759 | 166307 | 166307 |
